# Treg Cell Resistance to Apoptosis in DNA Vaccination for Experimental Autoimmune Encephalomyelitis Treatment

**DOI:** 10.1371/journal.pone.0049994

**Published:** 2012-11-14

**Authors:** Youmin Kang, Yuhan Sun, Jingyao Zhang, Wenjuan Gao, Jingjing Kang, Yongqiang Wang, Bin Wang, Guoliang Xia

**Affiliations:** 1 State Key Laboratory for Agro-Biotechnology, College of Biological Science, China Agricultural University, Beijing, China; 2 Department of modern Sciences & Technology, Agricultural University of Hebei, Baoding, China; 3 State Key Laboratory for Agro-Biotechnology, College of Veterinary Medicine, China Agricultural University, Beijing, China; 4 Key Laboratory of Medical Molecular Virology of MOH and MOE, Fudan University, Shanghai, China; Institute Biomedical Research August Pi Sunyer (IDIBAPS) - Hospital Clinic of Barcelona, Spain

## Abstract

**Background:**

Regulatory T (Treg) cells can be induced with DNA vaccinations and protect mice from the development of experimental autoimmune encephalomyelitis (EAE), a mouse model of multiple sclerosis (MS). Tacrolimus (FK506) has been shown to have functions on inducing immunosuppression and augmenting apoptosis of pathologic T cells in autoimmune disease. Here we examined the therapeutic effect of DNA vaccine in conjunction with FK506 on EAE.

**Methodology/Principal Findings:**

After EAE induction, C57BL/6 mice were treated with DNA vaccine in conjunction with FK506. Functional Treg cells were induced in treated EAE mice and suppressed Th1 and Th17 cell responses. Infiltrated CD4 T cells were reduced while Treg cells were induced in spinal cords of treated EAE mice. Remarkably, the activated CD4 T cells augmented apoptosis, but the induced Treg cells resisted apoptosis in treated EAE mice, resulting in alleviation of clinical EAE severity.

**Conclusions/Significance:**

DNA vaccine in conjunction with FK506 treatment ameliorates EAE by enhancing apoptosis of CD4 T cells and resisting apoptosis of induced Treg cells. Our findings implicate the potential of tolerogenic DNA vaccines for treating MS.

## Introduction

MS is a chronic inflammatory autoimmune disease of the central nervous system (CNS). EAE is an inflammatory demyelinating disease of the CNS and serves as the principle model for human MS [1]. EAE can be induced in rodents by immunization with myelin proteins, such as myelin basic protein (MBP), proteolipid protein (PLP), and myelin oligodendrocyte glycoprotein (MOG) or peptides [2], [3]. Much work has been focused on devising strategies to enhance therapeutic induction of Treg cells, which can be achieved by using DNA vaccine encoding autoantigens or derived peptides [4], [5], [6]. The induction of autoantigen-specific Treg cells can result in the local dampening of autoimmune processes even if the antigen specificities of the autoaggressive T cells are not known.

Apoptosis is an active process involved in many steps of development and maintenance of the immune system [7] and also required for the generation and maintenance of self-tolerance. Activated self-reactive T cells could undergo apoptosis in a variety of autoimmune diseases including EAE [8]. Thus the apoptosis of pathogenic CD4 T cells could contribute to the EAE therapy [9]. FK506 is a clinically used effective immunosuppressive agent and promoter of immunologic tolerance [10]. FK506 suppresses the activation of immune cells and production of IL-2 by T cells, which is considered to be responsible for its strong suppression of cellular immunity [10], [11]. However, limited information is available about the mechanism of FK506-induced immunosuppression. Evidence has accumulated that FK506 significantly augmented apoptosis of T cells [12], [13], [14], [15]. It was showed that FK506 enhanced dexamethasone (DEX) -induced apoptosis of T cells *in vivo* and *in vitro* [13]. FK506 treatment significantly augmented thymic apoptosis induced by anti-CD3 Ab administration *in vivo* and apoptosis of staphylococcal enterotoxin B (SEB) specific T cells [14]. It was reported that FK506 augmented T cell apoptosis of naive splenocytes which were activated by PMA and ionomycin *in vitro* and prevented spontaneously autoimmune pancreatitis [15]. These studies indicate that FK506-triggered apoptosis may represent a potential mechanism of the immunological tolerance achieved in FK506 treatment. In this study, we investigated the therapeutic effect of DNA vaccine in conjunction with FK506 on EAE. Our data showed that tolerogenic DNA vaccination ameliorated EAE by augmenting apoptosis of pathologic CD4 T cells and resisting apoptosis of induced Treg cells.

## Results

### The therapeutic effect of DNA vaccination on EAE

**Figure 1:**
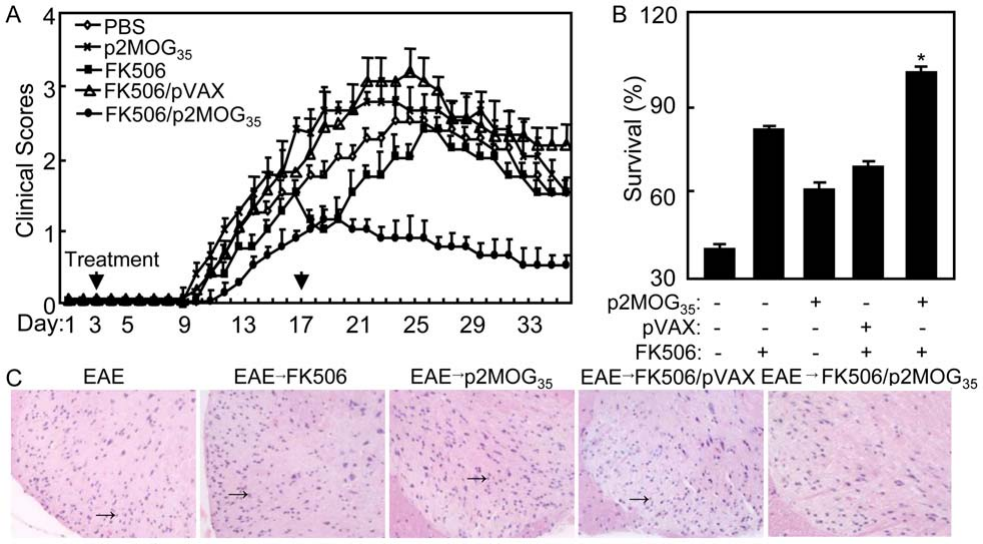
Therapeutic effect of tolerogenic DNA vaccine on EAE mice treatment. A. At day 3 and 17 after the mice induced for EAE, EAE mice were treated with FK506/p2MOG35 twice. The clinical scores of treated EAE mice were evaluated every day. B. Three months after the mice induced for EAE, the survival EAE mice were counted. C. At day 4 after the 2^nd^ treatment, spinal cords of EAE treated mice were prepared for histological analysis described in *Materials and Method*s (magnification, ×200). Solid arrows indicate infiltrating lymphocytes in spinal cords.

To test the effect of DNA vaccine in conjunction with FK506 on EAE treatment, EAE mice were treated and checked for clinic score daily. The clinic scores of EAE mice treated with p2MOG_35_/FK506 were the lowest than that in other groups (Fig. 1A). Three months later, the EAE mice treated with p2MOG_35_/FK506 were still alive. However, 60 percent of the nontreated EAE mice, 20 percent of EAE mice treated with p2MOG_35_ alone, 40 percent of EAE mice treated with FK506 alone and 30 percent of EAE mice treated with FK506 alone died (Fig. 1B). Less infiltration was observed in the p2MOG_35_/FK506 treated EAE mice while heavy lymphocyte infiltration into the spinal cord was found in the nontreated EAE mice, p2MOG_35_ treated EAE mice, FK506 treated EAE mice and pVAX/FK506 treated EAE mice(Fig. 1C).

### Immune tolerance restored in treated EAE mice

**Figure 2:**
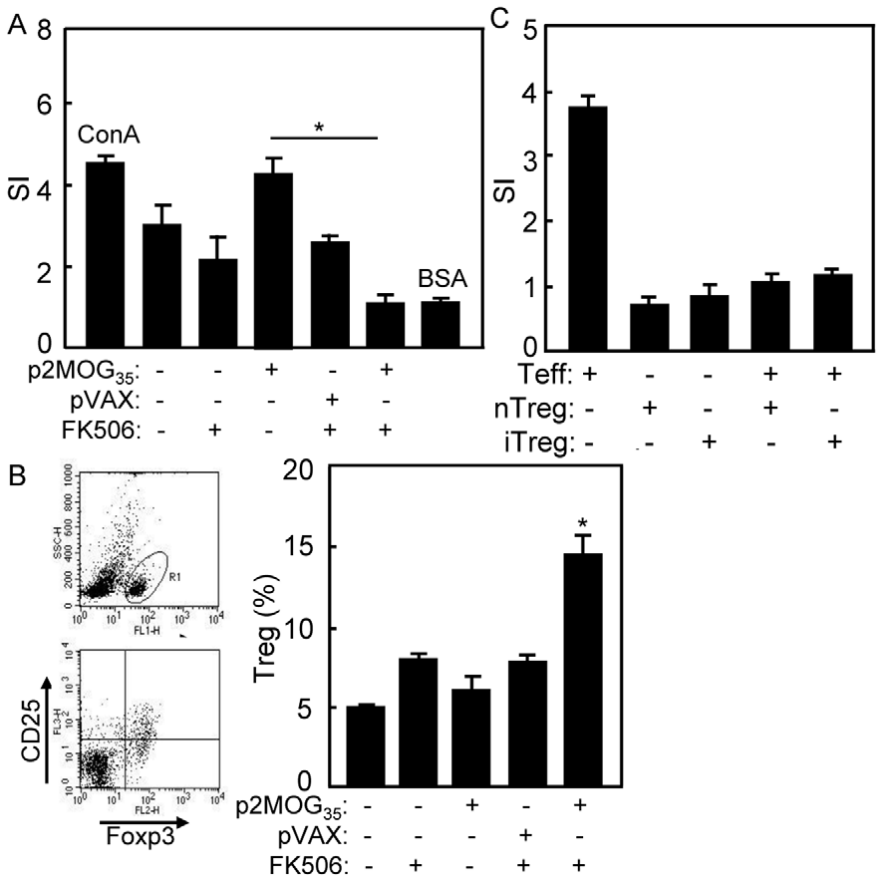
Immune tolerance was restored in treated EAE mice. A. At day 4 after the second treatment, T cell proliferation by MTT method was performed with MOG_35–55_ peptide as stimulation antigen, ConA as positive control and BSA as irrelevant antigen. B. At day 4 after the second treatment, the splenocytes of treated EAE mice were prepared for Treg cells analysis. Gating on CD4^+^ T cells, Treg cells (CD4^+^CD25^+^Foxp3^+^) were counted relatively to total CD4 cells by flow cytometry. C. Treg cells from naïve mice (nTreg) or FK506/p2MOG_35_ treated mice (iTreg) were co-culture with CD4^+^CD25^−^ T cells from naïve C57BL/6 mice respectively. Proliferation was tested by MTT method. Bar, mean and SD from 3 independent experiments, each using at least three mice per group (n = 3); *, p<0.05 between the indicated pairs.

To test the effect of tolerogenic DNA vaccine treatment on T cells, T cell responses of treated EAE mice were compared. T cell proliferation was performed as shown in Figure 2A. Since self-antigen specific responses were already activated in EAE mice, p2MOG_35_ boosted strong proliferative response of T cell in spleens of EAE mice. However, p2MOG_35_/FK506 significantly suppressed T cell proliferation compared with p2MOG_35_ group (*p*<0.05). The proliferative responses of T cells were less activated in FK506 alone and pVAX/FK506 treated mice as expected. For induction of Treg cells test, splenocytes from p2MOG_35_/FK506 treated EAE mice were stained with anti-CD4, ani-CD25 and anti-Foxp3 mAbs and analyzed by FACS. Gating on CD4^+^ cells, Treg cells (CD4^+^ CD25^+^ Foxp3^+^) were counted relatively to total CD4 cells (Fig. 2B). The results showed an elevated count of Treg in p2MOG_35_/FK506 treated EAE mice compared with other groups (*p*<0.05) (Fig. 2B). To test whether the induced Treg cells are functional, suppression assay was performed. Treg cells from mice treated with p2MOG_35_/FK506 significantly inhibited the proliferation of responsor T cells in co-culture (Fig. 2C). These results revealed the capability of p2MOG_35_/FK506 to restore tolerance in EAE mice.

**Figure 3:**
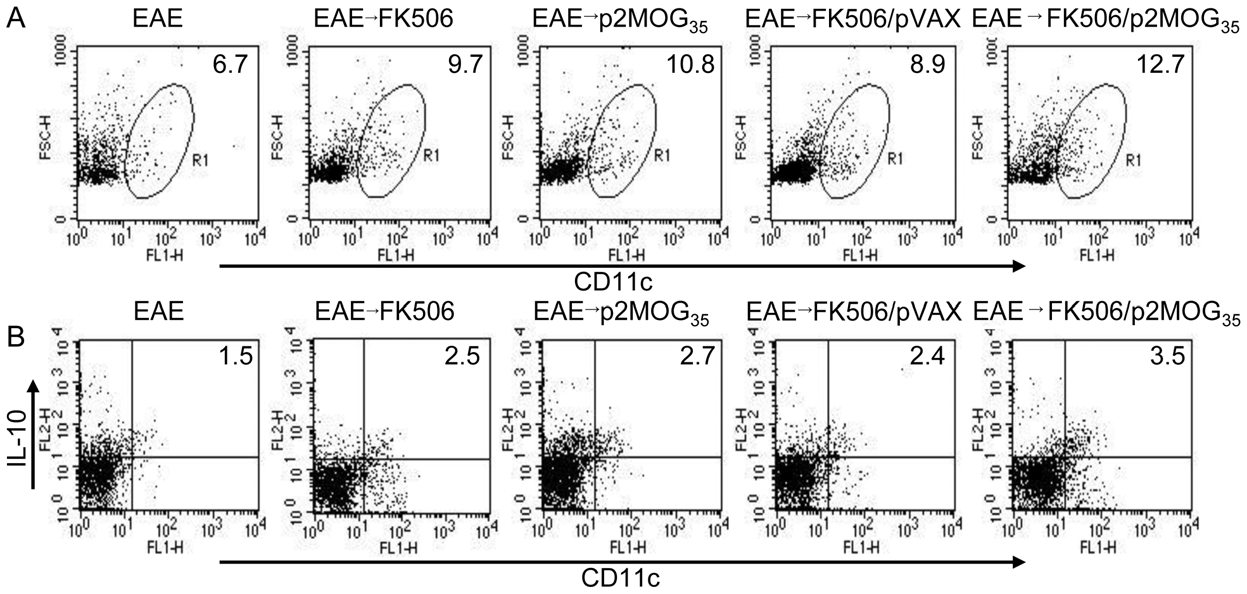
Tolerogenic DCs were stimulated in treated EAE mice. On day 3 after the second treatment, the splenocytes were prepared and intracellularly stained with anti-CD11c and anti-IL-10 mAbs. A. CD11c^+^ cells were counted relatively to total splenocytes by flow cytometry. B. CD11c^+^IL-10^+^ cells were counted relatively to total CD11c^+^ DC cells by flow cytometry. Shown in each panel is 1 of at least 3 experiments with similar results.

Treg cells are known to be induced by tolerogenic dendritic cells (DCs), whereas FK506 can suppress antigen presentation and prevent DC maturation *in vitro* [16], [17]. We therefore analyzed tolerogenic DC in EAE mice treated with p2MOG_35_/FK506. As shown in Figure 3, DCs (CD11c^+^) count in EAE mice treated with p2MOG_35_/FK506 was increased significantly compared with that in nontreated EAE mice (*p*<0.05), while DCs counts in EAE mice treated with p2MOG_35_, FK506 alone or pVAX/FK506 increased moderately. The expression of IL-10 in DC cells in EAE mice treated with p2MOG_35_/FK506 was increased significantly compared with other groups, suggesting that p2MOG_35_/FK506 treatment stimulated the production of tolerogenic DCs in EAE mice.

### Cytokines expression in CD4 T cells after EAE treatment

**Figure 4:**
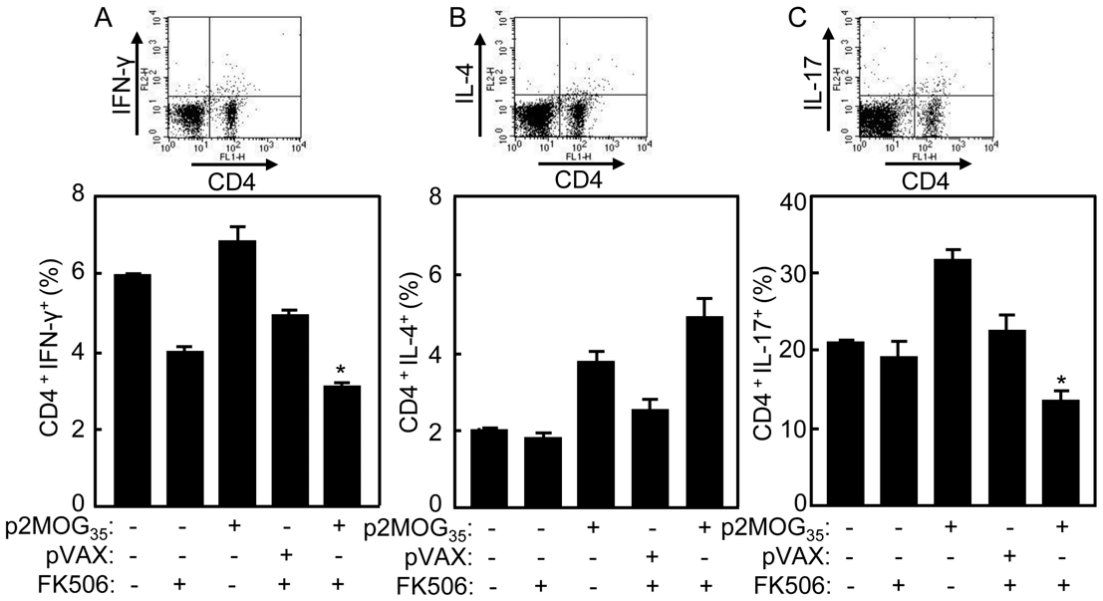
Suppression of Th1 and Th17 cell responses in treated EAE mice. At day 4 after the second treatment, the splenocytes of treated mice were prepared for intracellular staining. A. The splenocytes were intracellularly stained with anti-CD4 and anti-IFN-γ mAbs. CD4^+^IFN-γ^+^ T cells were counted relatively to total CD4 T cells by flow cytometry. B. The splenocytes were intracellularly stained with anti-CD4 and anti-IL-4 mAbs. CD4^+^IL-4^+^T cells were counted relatively to total CD4 T cells by flow cytometry. C. The splenocytes were intracellularly stained with anti-CD4 and anti-IL-17 mAbs. CD4^+^IL-17^+^ T cells were counted relatively to total CD4 T cells by flow cytometry. Bar, mean and SD from 3 independent experiments, each using at least three mice per group (n = 3); *, p<0.05 between the indicated pairs.

Cytokines play an important role in immune polarization. To test the cytokine profiles in treated EAE mice, splenocytes were prepared and intracellularly stained with anti-CD4, anti-IFN-γ, and anti-IL-4 or anti-IL-17 mAbs. CD4^+^IFN-γ^+^, CD4^+^IL-4^+^, and CD4^+^IL-17^+^ T cells were counted relatively to total CD4^+^ T cells by flow cytometry (Fig. 4). As shown in Figure 4A, the p2MOG_35_/FK506 treated EAE mice had fewer IFN-γ^+^ T cells than other groups (*p*<0.05), especially when compared to the p2MOG_35_ alone or nontreated EAE mice. For IL-4 expression, p2MOG_35_/FK506 and p2MOG_35_ alone treated EAE mice had more IL-4^+^ T cells than other groups (*p*<0.05) (Fig. 4B). For IL-17 expression, p2MOG_35_/FK506 treated EAE mice had fewer IL-17^+^ T cells than other groups (*p*<0.05). However, p2MOG_35_ alone treated EAE mice had the highest expression of IL-17 (Fig. 4C). These results suggested that p2MOG_35_/FK506 treatment impaired Th1 and Th17 cells responses.

### Accumulative Treg cells in spinal cord after EAE treatment

**Figure 5:**
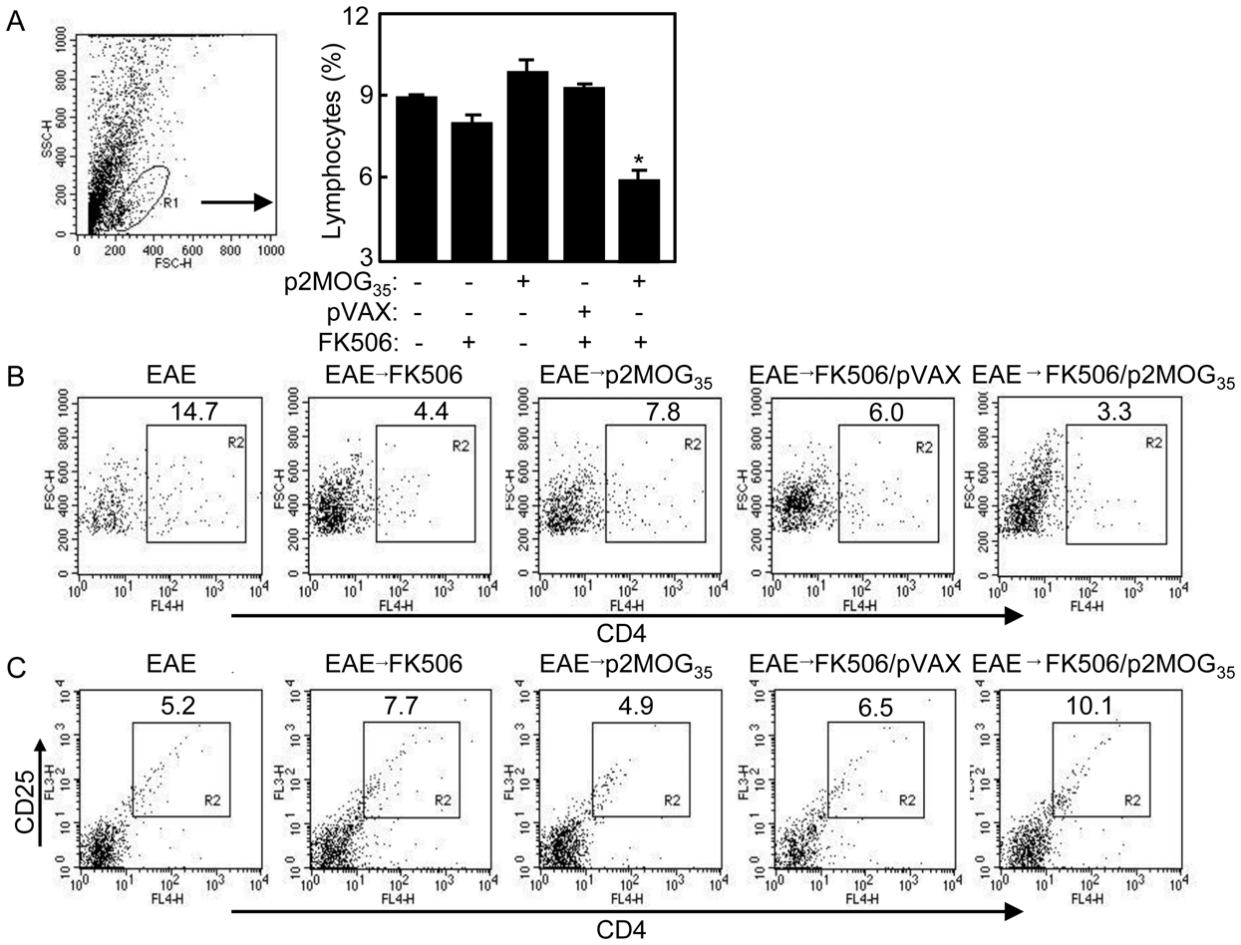
Tolerance was induced in spinal cord of treated EAE mice. On day 4 after the second treatment, the spinal cords were digested by collagenase D and DNaseI and centrifuged with Percoll Gradient. A. Infiltrating lymphocytes (gate-R1) were counted relatively to total spinal cord cells. Bar, mean and SD from 2–4 independent experiments, each using at least three mice per group (n = 3); *, p<0.05 between the indicated pair. B. CD4^+^ T cells were counted relatively to total Infiltrated lymphocytes by flow cytometry. C. CD4^+^CD25^+^ Treg were counted relatively to total CD4^+^ cells by flow cytometry. Shown in each panel is 1 of at least 3 experiments with similar results.

To analyze the composition of the infiltrating lymphocytes, the spinal cords were flushed, digested, and separated by Percoll gradient centrifugation. Mononuclear cells from the interphase were resuspended in culture medium (gate-R1, Fig. 5A), lymphocytes (Fig. 5A), CD4^+^ T cells (Fig. 5B) and CD4^+^CD25^+^ Treg cells (Fig. 5C) were analyzed by FACS. As shown in Figure 5A, less lymphocytes were noted in the spinal cord of EAE mice treated with p2MOG_35_/FK506 compared to other groups (*p*<0.05). EAE mice treated with p2MOG_35_, FK506 alone or pVAX/FK506 showed the fewer CD4 T cells in spinal cord. The percentage of Treg cells (CD4^+^CD25^+^) in spinal cord of p2MOG_35_/FK506 treated EAE mice were significantly increased compared with other groups (*p*<0.05). The percentage of Treg cells in EAE mice treated with FK506 alone or pVAX/FK506 were higher than p2MOG_35_ treated and nontreated groups. These results showed that immune tolerance was also induced in spinal cord of treated EAE mice.

### Apoptosis augmented in CD4 T cells and resisted in Treg cells after EAE treatment

**Figure 6:**
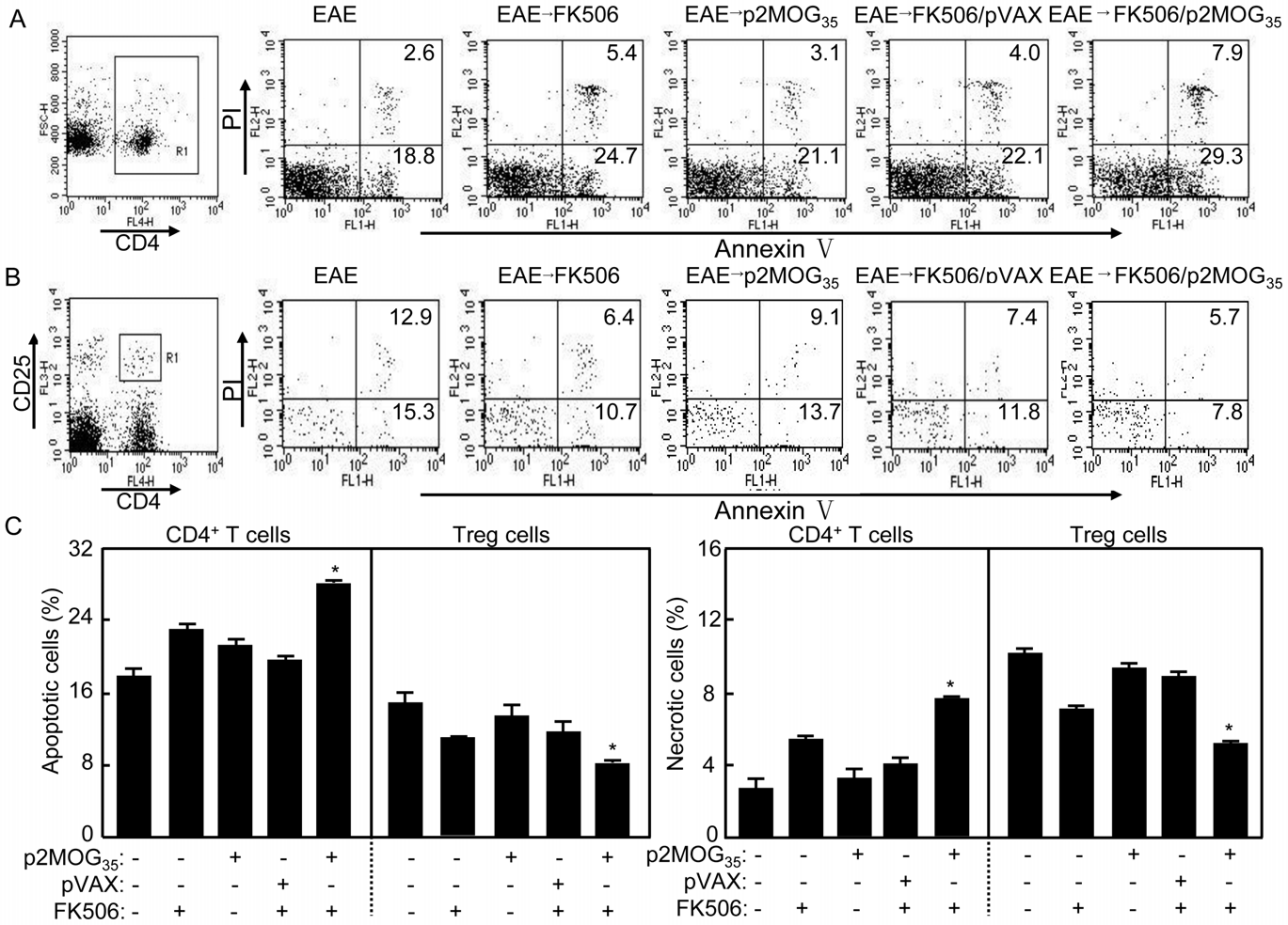
CD4 T cells augmented apoptosis and Treg cells resisted apoptosis in treated EAE mice. A. On day 4 after the second treatment, the splenocytes were prepared for apoptosis test. A. The splenocytes gating on CD4^+^ T cells were analyzed for annexinV/PI by flow cytometry. Shown in each panel is 1 of at least 3 experiments with similar results. B. Gating on CD4^+^CD25^+^ Treg cells, the samples were analyzed for apoptosis by flow cytometry. Shown in each panel is 1 of at least 3 experiments with similar results. C. Summary of the percentage of apoptotic and necrotic CD4^+^ T cells and Treg cells. Bar, mean and SD from 3 independent experiments, each using at least three mice per group (n = 3); *, p<0.05 between the indicated pairs.

FK506 was reported to augment apoptosis of activated T cells [12], [13], [14], [15]. To test whether FK506 as adjuvant of DNA vaccine can induce apoptosis of T cells for EAE treatment, splenocytes were prepared and stained for apoptosis analysis. Gating on CD4^+^ T cells, the apoptotic CD4 T cells (CD4^+^AnnexinV^+^/PI^−^) were counted relatively to total CD4 T cells (Fig. 6A). The percentage of apoptotic CD4 T cells was significantly increased in mice treated with p2MOG_35_/FK506 than other groups (*p*<0.05). Interestingly, when Treg cells were gated for apoptosis analysis (Fig. 6B, 6C), the percentage of apoptotic Treg cells (CD4^+^CD25^+^ AnnexinV^+^/PI^−^) was significantly decreased in mice treated with p2MOG_35_/FK506 when compared with other groups (*p*<0.05). Consistently, the necrotic CD4^+^ T cells (CD4^+^AnnexinV^+^/PI^+^) was significantly increased in mice treated with p2MOG_35_/FK506 when compared with other groups (*p*<0.05) whereas the percentage of necrotic Treg cells (CD4^+^CD25^+^AnnexinV^+^/PI^+^) from p2MOG_35_/FK506 treated mice was significantly lowered compared with other groups (*p*<0.05) (Fig. 6C). The CD11c^+^ cells were also tested for apoptosis analysis, but there are no differences among all groups (Data not shown).

## Discussion

Apoptosis is required for maintenance of self-tolerance and the apoptosis of activated self-reactive T cells contributes to EAE treatment [8]. The present study showed that activated splenic CD4 T cells from p2MOG_35_/FK506 treated EAE mice were liable to apoptosis while Treg cells resisted to apoptosis. Importantly, DNA vaccination had the potential to restore self-antigen tolerance and alleviate clinical EAE severity, implicating that DNA vaccination could be used as therapeutic treatment.

DNA vaccinations were tried before for EAE treatment. Vaccination with DNA encoding the MOG_91–108_ suppressed MOG_91–108_-induced rat EAE which was dependent on inclusion of CpG DNA and was associated with early induction of IFN-β [18]. Induction of Treg cells could be achieved through DNA vaccinations [19]. A DNA vaccine encoding for a peptide from the FR3 region of the TCR Vβ8.2 effectively induced Treg cells and prevented the development of EAE [20]. Suppression of clinical EAE was associated with expansion of Treg in the periphery when MOG-DNA vaccination was used for EAE treatment [21]. FK506, as immunosuppressive agent, effectively promoted immunologic tolerance [19]. We previously found that naïve mice immunized with p2MOG35/FK506 induced Treg cells and effectively prevented EAE [4]. Here, the relevance of these findings was established for EAE treatment, in which p2MOG35/FK506 also augmented the induction of Treg cells and resulted in amelioration of EAE. The tolerogenic DCs from EAE mice treated with p2MOG35/FK506 were increased which was similar to that in naïve mice immunized with p2MOG35/FK506 for EAE prevention. Since the autoreactive responses were already activated in EAE mice, the p2MOG_35_ treatment did not stimulated the induction of Treg cells which was consistent with the result of p2MOG_35_ vaccination for EAE prevention.

Cytokines play important roles in immune cell differentiation and polarization into functional subtypes and in directing their biological functions in autoimmunity [22]. EAE was previously considered to be a purely IL-12-driven Th1-mediated autoimmune disease [1]. However, recent data have established that IL-17-producing Th17 cells play a pivotal role in the pathogenesis of EAE [9], [23]. DNA vaccination could inhibit the EAE through induction of Treg cells or the induction of immune deviation [24]. DNA vaccine encoding MOG_91–108_ reduced proinflammatory Th17 cell responses and suppresses MOG_91–108_-induced rat EAE, but the expression of Foxp3 and Tumor Growth Factor (TGF)-β1, which are associated with Treg cells, was not enhanced [25]. DNA vaccination encoding full MOG gene dampened self antigen-specific proinflammatory Th1 and Th17 immune responses and induced expansion of Treg cells in the periphery [21]. Co-administration of a DNA vaccine encoding IL-10 together with a plasmid encoding a MBP_68–86_ rapidly amplified Treg-mediated response and suppressed an ongoing EAE [26]. Codelivery of IL-4 gene and a DNA vaccine encoding PLP_139–151_ caused Th2 immune response and protected immunity against EAE [27]. In addition, DNA vaccination may activate different effectors including cytotoxic responses and autoantibody production which can lead to different effect of DNA vaccination on EAE prevention and treatment [19]. We previously found naïve mice immunized with p2MOG35/FK506 markedly impaired the Th17/Th1 responses and increased Th2 responses for EAE prevention [4]. In present study, p2MOG35/FK506 treatment of EAE mice suppressed Th17/Th1 cells responses consistently while Th2 cells responses was not enhanced. Since the autoreactive responses have already been activated in EAE mice, p2MOG_35_ treatment boosted stronger Th17 responses than that in nontreated EAE mice. However, p2MOG_35_ vaccination for EAE prevention stimulated weaker Th17 responses which was less than in control mice, and resulted in amelioration of EAE. Thus, different mechanisms may account for the tolerance induction and immune deviation observed in EAE prevention and EAE therapy.

Apoptosis is a signal-dependent suicide form of cell death and is required for the generation and maintenance of self-tolerance [28]. Autoreactive cells are removed by apoptosis during the process of lymphocyte development, selection, and education to prevent autoimmunity [29]. Dysregulation of apoptosis is a central defect in diverse murine autoimmune diseases. Mature T cells undergo apoptosis when they are persistently exposed to TCR stimulation [30]. Accumulation of activated autoreactive T cells at the site of inflammation has been reported in a variety of autoimmune diseases including EAE [1]. Thus, it is possible that these pathogenic T cells can undergo apoptosis before clonal expansion. As an immunosuppressant, FK506 has been reported to augment thymic apoptosis by anti-CD3 Ab administration *in vivo* [14]. The enhancement of FK506-induced apoptosis of SEB-activated splenic T cells is mediated by down-regulation of Bcl-X(L) expression on these cells [12]. Pretreatment with FK506 significantly augmented DEX-induced thymocyte apoptosis *in vivo* and enhanced DEX-induced apoptosis of human peripheral blood T cells *in vitro* [13]. FK506 treatment on autoimmune pancreatitis mice augmented apoptosis of activated T cells but not resting cells which suggest that FK506 may act selectively on activated T cells at the site of inflammation [15]. Evidence has accumulated that FK506-triggered apoptosis may represent a potential mechanism of the immunological tolerance achieved by FK506 treatment. In this study, we demonstrated that EAE mice treated with p2MOG_35_/FK506 enhanced apoptosis of CD4 T cells indicating the assistance of FK506 to DNA vaccine for EAE treatment.

Treg cell dependent suppression and apoptosis of immune effector cell are both essential in establishing and maintaining peripheral tolerance. Failure in either process can result in an overshooting immune response and foster the development of autoimmunity. Treg cells have been shown to act through a variety of mechanisms [31]. Naive Treg cells present variable sensitivities to apoptosis while activated Treg cells are less sensitive to apoptosis than cytotoxic effector subsets [32]. Treg and T effector cells' sensitivity to Fas-dependent apoptosis could be modified during the course of an immune response for maintaining an equilibrium between Treg and T effector cells [33]. Comparation with conventional T cells, Treg cells were much more resistant to apoptosis via TCR, but were sensitive to the purinergic receptor P2X_7_-stimulated cell death [34]. In autoimmune thyroid disease (AITD) patients, intrathyroidal Treg cells were decreased in response to apoptosis which may contribute to the incomplete regulation of autoreactive T cells in AITD [35]. In SLE patients, the abnormal apoptosis of Treg cells was induced which may be one of the pathogenic mechanisms of SLE [36]. In this study, our data show that the induced Treg cells from tolerogenic DNA vaccine treated EAE mice were significantly resistant to apoptosis than in control groups. Thus, sensitivity to apoptosis of Treg cells maybe important in immune tolerance that affects the immune equilibrium in activation and termination of immune activity.

In summary, our results demonstrated that a DNA vaccine encoding self-antigen peptide combined with FK506 could effectively augment apoptosis of pathogenic CD4 T cells and decrease apoptosis of Treg cells for EAE treatment. Our findings provide an effective method and reveal the function of Treg cells in restoring tolerance for MS treatment.

## Materials and Methods

### Animals and reagents

Female C57BL/6 mice at 6–8 weeks of age were purchased from Animal Institute of Chinese Medical Academy (Beijing, China). All animal protocols (#20120101) were approved by the Animal Welfare Committee of China Agricultural University and housed with pathogen-free food and water under 12 h light-cycle conditions. FK506 was from Astellas Ireland Co., Ltd. All antibodies for flow cytometry analysis were from eBioscience. The plasmid p2MOG_35_ encoding 2 copies of MOG35–55 DNA sequences and pVAX plasmid were prepared as described previously [4]. MOG_35–55_ peptide (MEVGWYRSPFSRVVHLYRNGK) was synthesized by GLBiochem Co., Ltd. (Shanghai, China). All antibodies for flow cytometry analysis were from eBioscience.

### EAE induction and treatment

EAE was induced by injecting the mice subcutaneously (into the flanks) with 100 µl of an emulsion containing 200 µg of MOG_35–55_ peptide (MEVGWYRSPFSRVVHLYRNGK) and 250 µg of *M. tuberculosis* extract H37 Ra (Difco) in incomplete Freund adjuvant. In addition, the mice received 200 ng of pertussis toxin (List Biological Laboratories via Cedarlane Ltd.) intraperitoneally (i.p.) at day 0 and 2. Clinical scores of EAE were assessed according to the following scale: 0, no signs of disease; 1, loss of tone in the tail; 2, hind limb paresis; 3, hind limb paralysis; 4, tetraplegia; 5, moribund [19].

At day 3 and 17 after the mice induced for EAE, mice (n = 5) were co-injected with FK506 (10 µg/mouse) and p2MOG_35_ plasmid (100 µg/mouse) into the hind leg muscles. This regimen was given twice in a 2 week interval.

Female C57BL/6 mice were immunized with incomplete Freund's adjuvant and MOG_35–55_ peptide twice in a 2-week interval. At day 4 after the 2nd immunization, the splenocytes were prepared as responders for suppression assay.

### T cell proliferation and suppression assays

Three mice from each group were sacrificed and single lymphocyte suspensions were prepared from the spleen at day 7 after the second treatment. T cell proliferation was performed with MOG_35–55_ peptide as antigen stimulation as described previously [37].

CD4^+^CD25^−^ T (Teff) cells from the spleen of C57BL/6 mice immunized with IFA/MOG_35–55_ peptide were enriched via negative selection by magnetic cell sorting (Miltenyi Biotec, Auburn, CA), following manufacturer's protocols, and used as responders. CD4^+^CD25^+^ T cells from EAE mice treated with p2MOG_35_/FK506 were enriched via positive selection by magnetic cell sorting and used as suppressors (iTreg), while CD4^+^CD25^+^ T cells from naïve C57BL/6 mice were used as controls (nTreg). CD11c^+^ cells were sorted by magnetic cell sorting (Miltenyi Biotec, Auburn, CA) from the spleens of naïve C57BL/6 mice and used as stimulators. The responders (0.5×10^6^ cells/well) were co-cultured with the suppressors (0.25×10^6^ cells/well), stimulators (0.5×10^6^ cells/well), and MOG_35–55_ (10 µg/ml) in U-bottom 96-well plates for 3 days at 37°C. The proliferation of the responder T cells was determined by the MTT method described before [37].

### Intracellular staining for flow cytometry analysis

For Treg cell analysis, splenocytes from treated mice were stained with anti-CD4, anti-CD25, and anti-Foxp3 mAbs, and Treg cells were counted relative to total CD4 cells by flow cytometry as described before [37]. For DCs analysis, splenocytes from treated mice were stained with anti-CD11c, anti-IL-10, and IL-10 expression of DCs were counted relative to total CD11c cells by flow cytometry as described before [37]. For intracellular cytokine staining, cells were treated as described previously [37] and stained with anti-CD4-FITC, fixed (1% paraformaldehyde), permeabilized (0.5% Tween 20), and intracellular immunostained with anti-IFN-γ-PE, anti- IL-4-PE, and anti-IL-17-PE. The cells were analyzed with a FACScalibur and the Cell Quest Pro Software (BD Bioscience). The result was presented as the percentage of cytokine positive cells relative to total CD4 T cells.

### Infiltrating lymphocytes in spinal cords

After 24 days of EAE induction, the spinal cord was flushed with PBS by hydrostatic pressure and digested by collagenase D (2.5 mg/ml, Roche Diagnostics) and DNaseI (1 mg/ml, Sigma) at 37°C for 45 min. Mononuclear cells were isolated by passing the tissue through a cell strainer (70 mm), followed by a Percoll gradient (70%/37%) centrifugation. Mononuclear cells were removed from the interphase, washed and resuspended in culture medium for further analysis. Infiltrating lymphocytes (gate-R1) in the spinal cord were stained with anti-CD4-FITC and/or anti-CD25− PECy5. CD4^+^ T cells were counted relatively to total spinal cord cells. The cells were analyzed with a FACScalibur and the Cell Quest Pro Software (BD Bioscience).

CD4^+^CD25^+^ Treg in the spinal cord were counted relatively to total CD4^+^ cells by flow cytometry.

### Histology analysis

At day 7 after the second treatment, spinal cords of treated EAE were collected and fixed in Bouin's solution (71.4% saturated picric acid, 9.5% formaldehyde, and 4.8% glacial acetic acid) for 24 h before being embedded in paraffin. Serial sections of 5 µm thickness were cut and stained with hematoxylin and eosin (H&E).

### Apoptosis detection

At day 7 after the second treatment, splenocytes from EAE mice in each group were prepared for apoptosis detection. Cells were first stained with anti-CD4-FITC, anti-CD4-APC/anti -CD25-PECy5 or anti-CD11c-APC, and then were examined for apoptosis by using Annexin V-FITC Apoptosis Detection Kit (BioVision, USA). CD4^+^ T cells and CD4^+^CD25^+^ Treg cells were gated for apoptosis analysis.

### Statistic analysis

For multiple-group analysis, ANOVA and the Bonferroni test were used. Differences are considered significant if p<0.05 and highly significant if p<0.01.
